# Severe Hypoxemia Due to Elongation and Shunting of Patent Foramen Ovale After Emergent Repair of Thoracic Aortic Dissection

**DOI:** 10.1016/j.jscai.2024.102171

**Published:** 2024-06-18

**Authors:** Gael Charbonne, Nicholas Whitmore, Damian Valencia, Raja Amir Nazir, Nathaniel Dittoe

**Affiliations:** a Department of Internal Medicine, Kettering Health Main Campus, Kettering, Ohio; b Department of Interventional Cardiology, Kettering Health Main Campus, Kettering, Ohio; c Department of Cardiovascular Disease, Kettering Health Main Campus, Kettering, Ohio

**Keywords:** critical care, patent foramen ovale, persistent postoperative hypoxemia, thoracic aortic dissection, transcatheter patent foramen ovale closure

## Abstract

We document the elongation and shunting of a patent foramen ovale (PFO) after thoracic aortic dissection repair in a 63-year-old man. Initially, a presurgical echocardiogram showed insignificant PFO shunting; however, severe hypoxemia and inability to extubate after thoracic aortic dissection repair necessitated further investigation. A repeat transesophageal echocardiogram after cardiothoracic surgery revealed significant PFO elongation with bidirectional shunting. Subsequent urgent transcatheter PFO closure markedly improved oxygenation, allowing for successful weaning from mechanical ventilation. This case highlights the importance of recognizing dynamic PFO changes after thoracic surgery as a reversible cause of postoperative hypoxemia.

## Introduction

Patent foramen ovale (PFO), a remnant in the interatrial septum, is present in about 25% to 30% of adults and is typically considered of little clinical importance under normal physiological conditions^1^; however, in rare instances involving dynamic intrathoracic changes during surgery or positive pressure from mechanical ventilation, a previously insignificant PFO can lead to a significant right-to-left shunt, resulting in hypoxemia.^2^^,^^3^ This case report illustrates a pronounced shift in PFO behavior due to cardiac manipulation during the emergent repair of an ascending aortic dissection using the Bentall procedure.

## Case description

**Figure 1:**
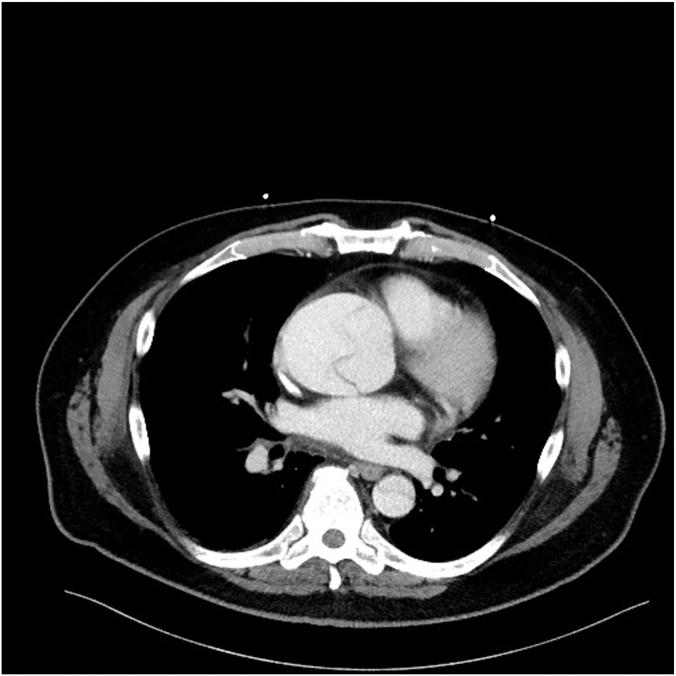
**CT angiography of the chestand axial planeshowing dissection of the ascending aorta**.

A 63-year-old man with a history of Crohn's disease, hyperlipidemia, and hypertension presented to the emergency department with chest pain and dizziness lasting several hours. The patient described the chest pain as anterior chest pressure radiating to the right arm, accompanied by nausea, dizziness, and lightheadedness. Acute coronary syndrome was quickly ruled out based on an electrocardiogram negative for ischemic changes and minimally elevated troponin levels. Further workup, including routine labs and imaging, showed hypokalemia, significantly elevated D-dimer, and elevated lactic acid. A CT angiography of the chest, abdomen, and pelvis revealed a Stanford type A aortic dissection extending into the brachiocephalic and subclavian arteries, with the distal flap reaching the celiac artery, left renal artery, and both common iliac arteries (Figure 1).

**Figure 2:**
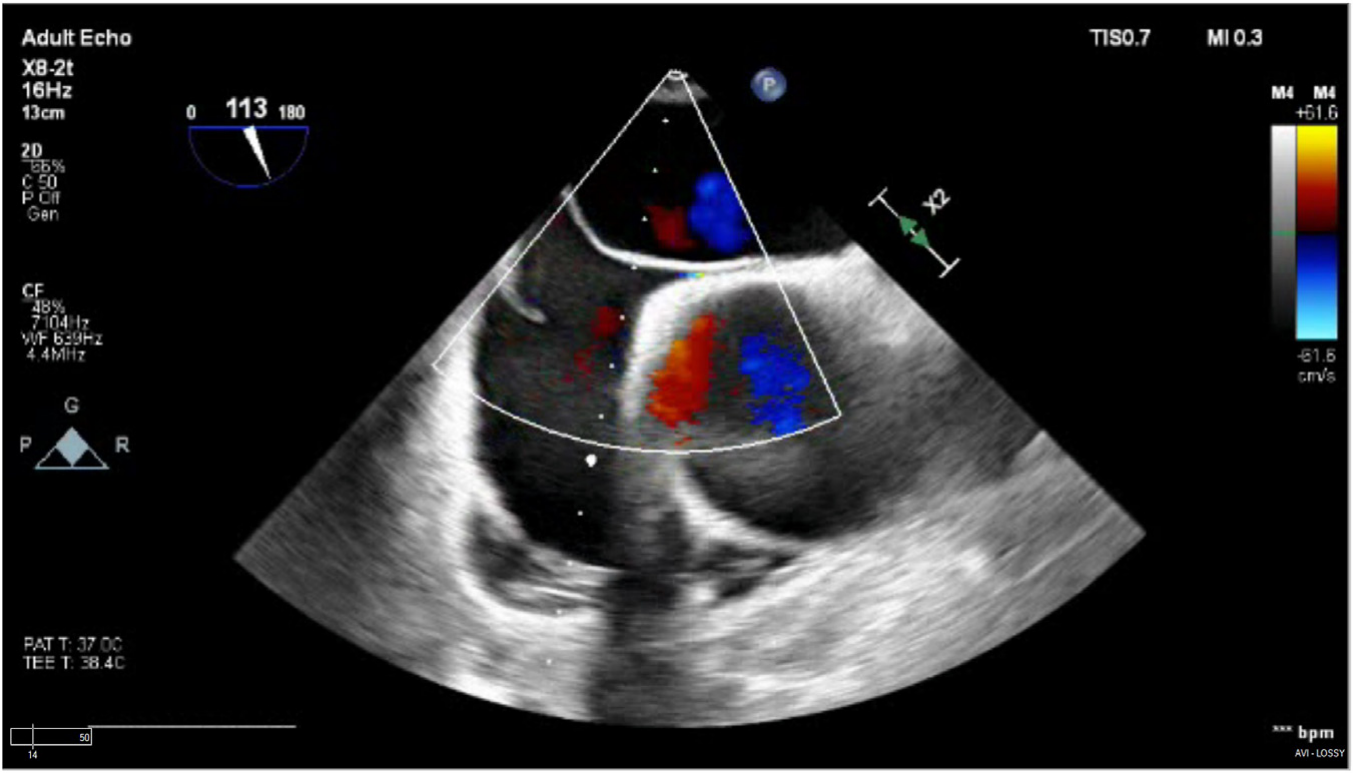
**Transoperative transesophageal echocardiogram conducted before the commencement of the Bentall procedure, depicting a short-tunneled patent foramen ovale with minimal interatrial flow**.

The patient underwent an emergent Bio-Bentall procedure under general anesthesia. Prior to the start of the operation, a transprocedural transesophageal echocardiogram (TEE) was conducted, confirming the aortic dissection and revealing a root aneurysm exceeding 6 cm with torrential aortic insufficiency. Notably, this preoperative TEE revealed a short-tunneled PFO with a weakly positive bubble study and minimal shunting (Figure 2). Upon opening the pericardium, hemorrhagic fluid was noted, though no overt rupture was found. For cardiopulmonary bypass, a cannula was placed in the axillary artery using a purse-string suture for antegrade flow, while a retrograde cannula was inserted into the right atrial appendage and dissection of the innominate and left carotid arteries facilitated the clamping necessary for antegrade cerebral perfusion. The aortic root was repaired with placement of a 25-mm INSPIRIS valve (Edwards Lifesciences) within a 26-mm Valsalva graft. Two mediastinal chest tubes and 1 right pleural chest tube were placed. The procedure concluded uneventfully, with no complications, and postprocedure TEE demonstrated a preserved ejection fraction but did not reevaluate the PFO at this stage.

**Figure 3:**
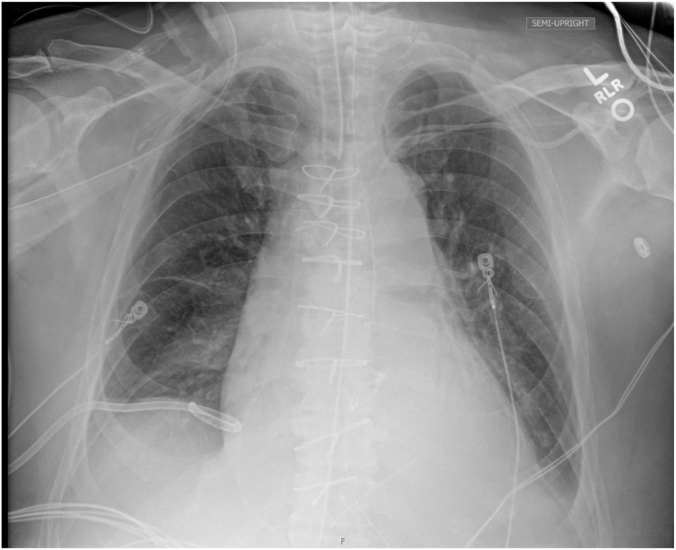
**Chestx-ray obtained on postoperative day 1, anteroposterior view, showing subtle infiltrates in the right lower lobe and trace left pleural effusion.** Mild left basilar atelectasis is also evident.

**Figure 4:**
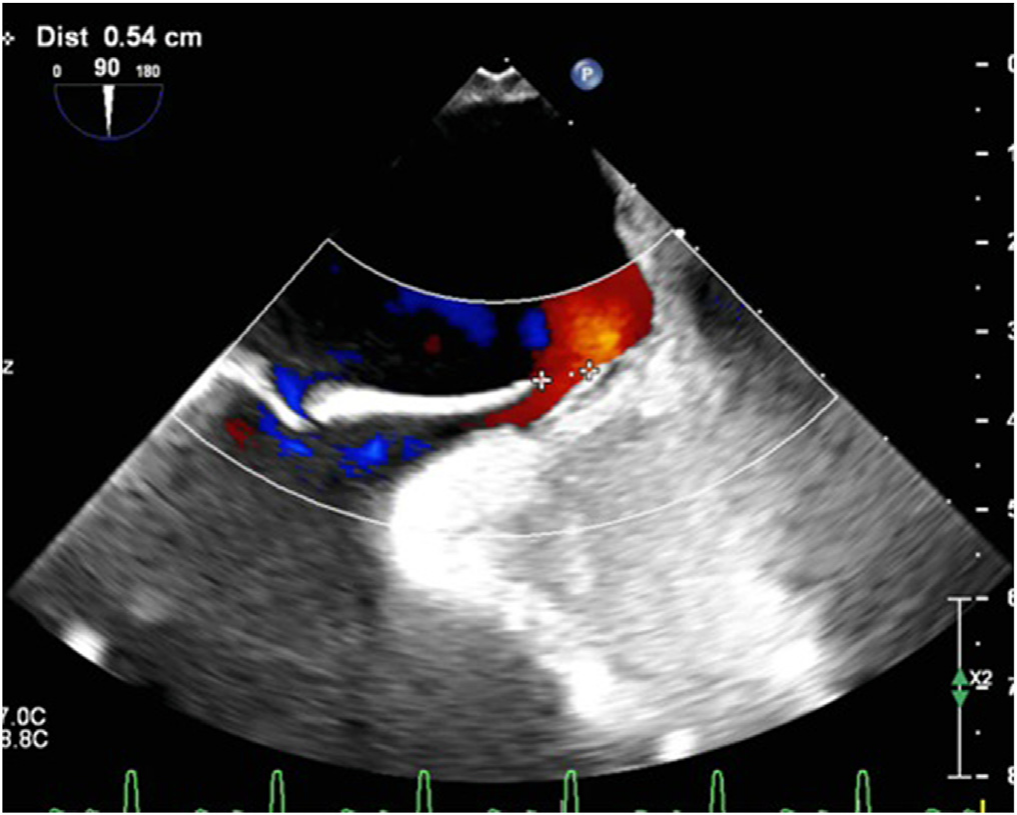
**Transesophageal echocardiogram on postoperative day 3 showing the long-tunnel patent foramen ovale with significant interatrial flow**.

The patient was then admitted to the intensive care unit (ICU) on mechanical ventilation with initial settings of pressure-regulated volume control mode, tidal volume of 450 mL, and FiO_2_ of 100% with positive end-expiratory pressure of 5 cm of H_2_O. Hemodynamic support was required using norepinephrine for the first 24 hours, after which the patient was successfully weaned off vasopressors. Given persistent hypoxemia on arterial blood gas and desaturation during attempts to wean the patient from ventilator by postoperative day 1, a chest x-ray was obtained, which revealed subtle right lower lobe infiltrate, a trace left pleural effusion, and mild left basilar atelectasis (Figure 3). By postoperative day 2, epoprostenol was started and the patient was paralyzed with nimbex, yet these interventions did not yield the expected improvement. On postoperative day 3, with the patient's hypoxemia deteriorating, positive end-expiratory pressure was increased to 10, and nitric oxide therapy was initiated as well. Subsequent chest x-ray showed no evidence of acute respiratory distress syndrome, pulmonary edema, pneumothorax, or worsening of atelectasis. At this critical juncture, a bedside TEE on postoperative day 3 was obtained, which revealed significant elongation and bidirectional shunting of the initially insignificant PFO (Figure 4). There was mild mitral valve regurgitation and the left ventricle ejection fraction was 50% to 55%.

**Figure 5:**
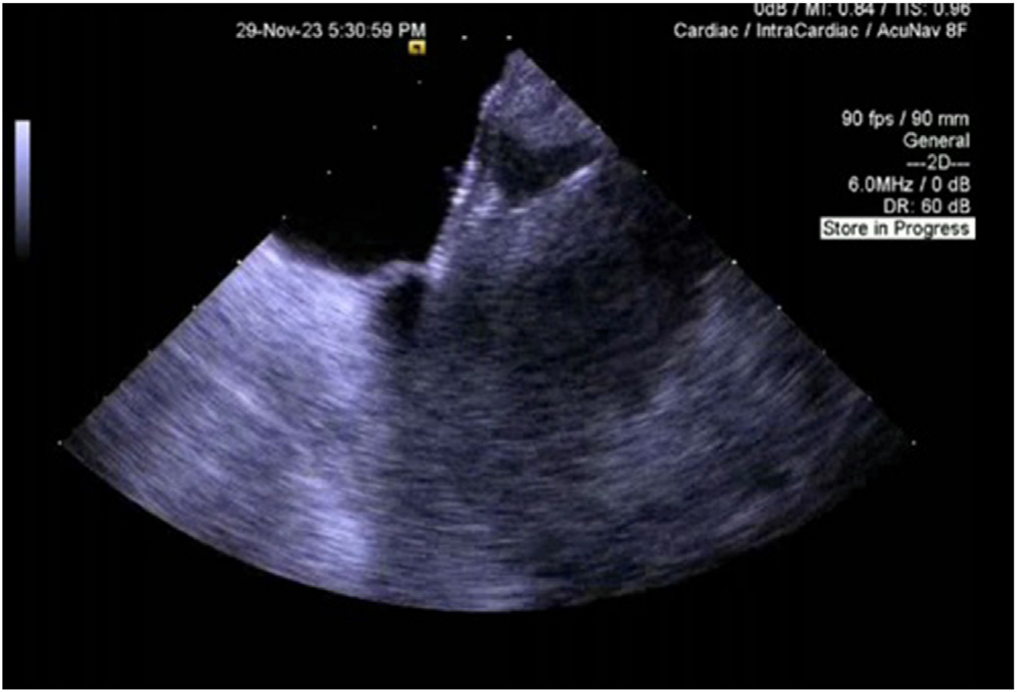
**Transesophageal echocardiogram during transcatheter patent foramen ovale closure showing Amplatzer Talisman 35-mm occluder device(Abbott)and successful closure of patent foramen ovale**.

An emergent transcatheter PFO closure was performed the same day using a 35-mm Amplatzer Talisman occluder device (Abbott), which led to a marked improvement in left atrial saturation from 74% to 94% immediately after deployment (Figure 5). Shortly after, the patient was successfully weaned off mechanical ventilation within the next 48 hours. He was discharged from the ICU and 5 days after the PFO closure, left the hospital breathing comfortably on room air, with plans for multidisciplinary follow-up on an outpatient basis.

## Discussion

Given the onset of resistant hypoxemia immediately following emergent thoracic aortic dissection repair, we hypothesized that cardiac manipulation during the Bentall procedure led to significant dynamic changes in the previously asymptomatic PFO. The patient required 100% FiO_2_ immediately upon transfer to the ICU, suggesting that the PFO elongation occurred during surgery. Although a transoperative TEE was performed at the start of the surgery, which showed a short PFO without shunting, the lack of reevaluation of the PFO at the conclusion of the surgery missed an opportunity to detect this complication earlier, potentially avoiding prolonged postoperative hypoxemia.

## Conclusion

It is important to consider the possibility of dynamic changes in previously insignificant PFO in patients who exhibit resistant hypoxemia and face challenges in weaning off mechanical ventilation after intrathoracic surgeries.^4^^,^^5^ In our case, the transcatheter closure of the PFO led to rapid resolution of hypoxemia. Additionally, this case underscores the need for special attention to be given to PFO evaluation at the beginning and conclusion of thoracic surgeries assisted by transoperative echocardiography.
